# High larvicidal efficacy of yeast-encapsulated orange oil against *Aedes aegypti* strains from Brazil

**DOI:** 10.1186/s13071-021-04733-2

**Published:** 2021-05-22

**Authors:** Bruno Gomes, Huarlen Ogélio, Fabiane Brant, Camila Jesus Pereira-Pinto, Michael J. Workman, Monique Costa, José Bento Pereira Lima, Ademir Jesus Martins, Marcelo Ramalho-Ortigao, Ravi Durvasula, Ivy Hurwitz, Mariana Rocha David, Fernando Ariel Genta

**Affiliations:** 1 Laboratório de Bioquímica E Fisiologia de Insetos, Instituto Oswaldo Cruz (IOC-Fiocruz), Rio de Janeiro, Brazil; 2 Laboratório de Mosquitos Transmissores de Hematozoários, Instituto Oswaldo Cruz (IOC-Fiocruz), Rio de Janeiro, Brazil; 3 Center for Global Health, University of New Mexico Health Sciences Center, Albuquerque, NM USA; 4 Department of Chemical and Biological Engineering, University of New Mexico, Albuquerque, NM USA; 5 Laboratório de Fisiologia E Controle de Artrópodes Vetores, Oswaldo Cruz Institute – Oswaldo Cruz Foundation (IOC-FIOCRUZ), Rio de Janeiro, Brazil; 6 Instituto Nacional de Ciência E Tecnologia Em Entomologia Molecular, Rio de Janeiro, Brazil; 7 Department of Preventive Medicine and Biostatistics, Uniformed Services University, Bethesda, MD USA; 8 Loyola University Stritch School of Medicine, Maywood, IL USA

**Keywords:** *Citrus sinensis*, Mosquito control, Arbovirus, Dengue, *Aedes aegypti*

## Abstract

**Background:**

Botanical substances such as essential oils (EOs) have demonstrated insecticidal properties and are a valid option for vector control. However, free EOs are unreliable as mosquito larvicides due their easy degradation by environmental exposure to ultraviolet light and higher temperatures. Here, we assessed the efficacy of a mosquito larvicide based on orange oil in a yeast-based delivery system against *Aedes aegypti* strains with different resistance status towards chemical neurotoxic insecticides. This larvicide preparation was physicochemically characterized in a previous report.

**Methods:**

Larvae of four *Ae. aegypti* strains from different regions of Brazil and different resistance profiles for deltamethrin (pyrethroid) and temephos (organophosphate) were tested against yeast-encapsulated orange oil (YEOO) in laboratory conditions for measurement of LC_50_ and LC_90_ values. The same assays were performed with the Belo Horizonte strain under environmental conditions (natural light and temperature). The resistance profiles of these strains were compared to the Rockefeller reference strain in all conditions.

**Results:**

YEOO was found to be a highly active larvicide (LC_50_ < 50 mg/L) against all *Ae. aegypti* strains tested in both laboratory conditions (LC_50_ = 8.1–24.7 mg/L) and environmental conditions with natural light and temperature fluctuation (LC_50_ = 20.0–49.9 mg/L). Moreover, all strains were considered susceptible (RR < 5) to YEOO, considering resistance ratios calculated based on the Rockefeller strain. The resistance ratios were only higher than 2.5 for LC_90–95_ of Belo Horizonte in the laboratory, probably due the higher heterogeneity associated with older egg papers (> 5 months).

**Conclusion:**

YEOO demonstrates high larvicidal activity against *Ae. aegypti* strains with resistant phenotypes for deltamethrin (PY) and temephos (OP). This larvicidal activity suggests the potential for the development of YEOO as an alternative intervention to synthetic insecticides in integrated vector management programs, for populations with resistance to commonly used insecticides.

**Graphic Abstract:**

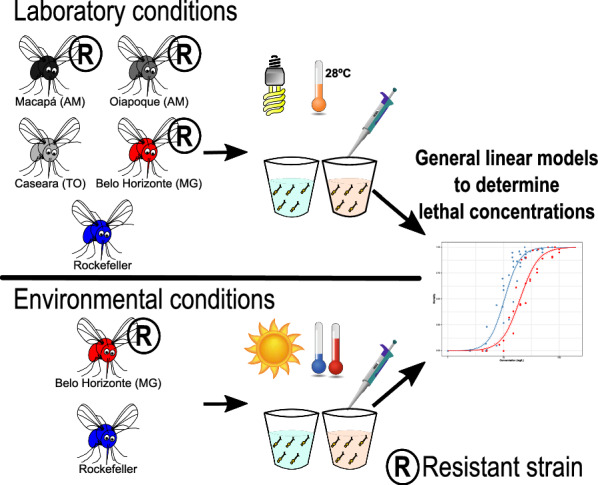

**Supplementary Information:**

The online version contains supplementary material available at 10.1186/s13071-021-04733-2.

## Background

Integrated vector management (IVM) is the rational decision-making process employed to optimize resources for disease vector control. This process aims to establish control strategies based on scientific knowledge about vectors and disease transmission to reduce the impact of vector-borne diseases. The implementation of IVM should include intersectional collaboration with multiple interventions based on entomological, social, and behavioral parameters, aiming to maximize the effectiveness of vector control programs [1]. In this framework, the development of novel tools for vector control is essential for health agencies and communities to enrich their IVM strategies and reduce their reliance on interventions based on chemical insecticides [2]. Historically, the intensive use of neurotoxic chemical insecticides in eradication campaigns for malaria and other diseases started in the 1940s [3]. Insecticides are still a crucial intervention to reduce the impact of mosquito-borne diseases worldwide. However, the toxicity of these compounds to the environment and the emergence of insecticide resistance are substantial limitations for maintaining sustainable vector control programs [4–6].

These limitations are particularly important for the mosquito *Aedes aegypti,* the primary mosquito vector in the transmission of arboviruses such as dengue, chikungunya, and Zika [7–9]. Over two million disability-adjusted life-years worldwide were attributed to *Aedes*-borne viruses in 2019 [10]. The continuous use of chemical insecticides to control *Ae. aegypti* is contributing to the emergence and spread of insecticide resistance [11]. The resistance to pyrethroids (PY) is widespread in *Ae. aegypti* from tropical regions, with multiple knockdown resistance alleles (*kdr* alleles) in the voltage-gated sodium channel already identified [11–14]. Resistance to the organophosphate (OP) temephos occurs in Brazil, French Guinea, and the Caribbean, associated with overexpression of multiple detoxifying enzymes including cytochrome P450 monooxygenases, glutathione S-transferases, and carboxy/cholinesterases [11, 15–18]. Insecticide resistance is particularly troublesome because pyrethroids, due to their low toxicity to humans, are widely used by vector control programs and communities to kill adult mosquitoes, and temephos is one of the few chemicals with application as a mosquito larvicide employed in drinkable water containers. In this context, the development of alternative tools is essential for the sustainability of IVM strategies targeting *Ae. aegypti* and arbovirus transmission [2].

Plant extracts and other botanicals (i.e. botanical active substances) have been critical in developing novel approaches for vector control [19]. Several secondary metabolites of plants are associated with insecticide properties (e.g. rotenoids, terpenoids) [20]. Botanicals can be effective as insecticides (e.g. pyrethrum extract that led to the development of synthetic pyrethroids [21]) and as repellents [22]. The term “botanicals” has a broad application that includes all substances obtained by processing plant materials using procedures such as pressing, milling, crushing, distillation, or extraction. These substances are complex mixtures of secondary plant metabolites that in some cases can be purified or concentrated to a single active element. They can also vary in properties such as their physical appearance (i*.e.* powders, liquid extracts) and solubility (i.e. hydrophilic, hydrophobic) [19].

Essential or volatile oils are hydrophobic liquids containing volatile substances extracted from plants. They present an oily appearance at room temperature and intense fragrance that varies according to their volatile components. Spectroscopic and chromatographic methods are used to characterize the chemical fingerprint of essential oils (EOs), and their primary components are generally characteristic of the plant tissue of origin. Still, other factors such as geography and climate [23] may also influence their composition. Several EOs are non-toxic to vertebrates at low concentrations, offering a wide range of applications in the cosmetics, food, and pharmaceutical industries. Though the larvicidal efficacy of EOs has been demonstrated against many mosquito species [24, 25], their integration as insecticides in IVM programs has yet to be realized [26]. The application of EOs as insecticides presents a few barriers regarding their production and deployment that the scientific community must overcome: (1) Due to their hydrophobicity, free EOs are not reliable for application in aquatic environments without disrupting the ecosystem, and (2) EOs are vulnerable to rapid degradation by ultraviolet (UV) radiation, temperature, and oxidation.

Our team has tackled this challenge in using EOs as mosquito larvicides by developing a new approach using a yeast-based delivery system [27]. The use of *Saccharomyces cerevisiae* (bakers’ yeast) as a biocompatible and biodegradable container for a variety of exogenous compounds has long been recognized in the pharmaceutical and food industries [28]. Additionally, the larvae of *Ae. aegypti* can easily digest the yeast cell wall [29–31]. In this context, our consortium developed a larvicide based on food-grade orange oil encapsulated in yeast cells that exhibited high activity (LC_50_ < 50 mg/L) against susceptible reference lineages of *Ae. aegypti* (i.e. Liverpool and Rockefeller) [27]. The yeast-encapsulated orange oil (YEOO) larvicidal activity was more consistent than that of orange oil reported in the literature, where many studies have shown negligible activity [32–34]. In the present study, we verify whether YEOO maintains its larvicidal activity when tested against *Ae. aegypti* strains with different resistance profiles to deltamethrin (PY) and temephos (OP) from Brazil. Our data also support the lack of cross-resistance mechanisms between chemical insecticides and the larvicide based on orange oil. Moreover, we also determine the variation in larvicidal activity under environmental conditions to assess the potential efficacy of YEOO under conditions found during the implementation of an IVM approach.

## Methods

### Larvicide synthesis

Larvicide was synthesized by encapsulation of *Citrus sinensis* EO (orange oil, California origin, Sigma-Aldrich, St. Louis, MO, USA) in *S. cerevisiae* (Red Star fresh baker’s yeast). The encapsulation method was adapted to existing processes as described by Workman et al*.* [27]. The components used in the synthesis were orange oil, fresh yeast, and water at a ratio of 1:5:16 by weight. Components were placed in a baffled flask and agitated overnight at 40 °C. The solution was then centrifuged, and the supernatant discarded. Residual, non-encapsulated oil was removed by washing, and the quantification of encapsulated orange oil was determined by high-performance liquid chromatography [27]. The YEOO was frozen and lyophilized for shipment, and larvicide aliquots were rehydrated to achieve a concentration of 50 mg/L orange oil in testing sites.

### Mosquito strains

Four separate strains of laboratory-reared* Ae. aegypti* from generations 2 to 12 were used in bioassays to determine the larvicidal activity of YEOO (Table 1). Mosquito strains from Caseara (TO; 09°16′40″ S, 49°57′21″ W), Oiapoque (AP; 3°50′39″ N, 51°49′55″ W), and Macapá (AP; 0°02′08″ N, 51°04′21″ W) were maintained at Oswaldo Cruz Institute in Fiocruz. The Belo Horizonte (MG) strain was established from 404 wooden pallets with ≈24,000 eggs of *Ae. aegypti.* This collection was performed during January 2018 in the northeastern area of Belo Horizonte city (19°49′13″ S, 43°55′06″ W), and eggs were hatched and maintained at Oswaldo Cruz Institute in Fiocruz. Each strain was identified as susceptible or resistant based on susceptibility to the insecticides deltamethrin (PY) and temephos (OP) as determined by dose–response bioassays on their initial generations F1–2 and literature data [35–38]. The information about the susceptibility/resistance profile of Belo Horizonte is only qualitative because we were unable to obtain information about insecticide screening for this strain. The information on temephos in the literature is more than 10 years old, and the current resistance status may be different, since this insecticide is no longer used by the local control agency [35]. For deltamethrin, the scenario is different; the control agency still sprays insecticides with deltamethrin (i.e. Fludora^®^ Fusion, Bayer, Germany) during disease outbreaks (e.g. dengue, chikungunya), and the mosquito population presents a high proportion of *Kdr* alleles [35, 38]. In addition, we used the Rockefeller strain as the reference for *Ae. aegypti* insecticide susceptibility (Table 1). Mosquito strains were labeled as resistant or susceptible according to the criteria recommended by the World Health Organization (WHO) for neurotoxic insecticides (RR < 5: not resistant; 5 < RR < 10: moderately resistant; RR > 10: highly resistant) [39].

**Table 1:** Information about strains of *Ae. aegypti*

Strain	State	Label	Generation	RR_50_ Deltamethrin	RR_50_ Temephos
Rockefeller	–	Reference		*NA*	*NA*
Caseara	TO	Susceptible	F9-F11	1.6	0.8
Oiapoque	AP	Resistant	F10-F12	143.9	21.8
Macapá	AP	Resistant	F9-F11	46.4	6.5
Belo Horizonte	MG	Resistant	F2-F3	Resistant	Resistant

Generation: the generations used for the bioassays with yeast-encapsulated orange oil. TO: Tocantins; AP: Amapá; MG: Minas Gerais. RR_50_: values calculated by dose–response bioassays using Rockefeller as reference strain for Caseara, Oiapoque, and Macapá, while qualitative information for Belo Horizonte is presented based on the literature [35, 38]. Resistant: strains were labeled as resistant with diagnostic dose assays carried out on initial generations (F1–2)

### Bioassays under laboratory conditions

Bioassays to evaluate the toxicity of YEOO for all Brazilian mosquito strains were carried out in controlled laboratory conditions (i.e. 28 ± 2 °C, 50–60%, 12/12 h regulated light with white fluorescent lamps) at Oswaldo Cruz Institute. The insectary temperature is maintained through the building's central air conditioning system, while humidity typically varies between 50 and 60%. We used an adapted version of the WHO-recommended protocol [40]. Eggs were hatched in filtered tap water under laboratory conditions with fish food (TetraMin, Tetra, Spectrum Brands Company, WI, USA) provided ad libitum (approx. 250 mg/day for 2000 larvae)*.* Groups of 20 to 25 early third-instar larvae (≈3 days after hatching) were placed separately into plastic cups. The larvicidal action is dependent on larval feeding behavior, and the early third-instar larval stage seems to be better for evaluating larvicides dependent on this behavior [27]. Each assay was carried out at ten concentrations with multiple subsets per concentration, and concentrations were adjusted to achieve 5% to 95% mortality for each condition. For the *Ae. aegypti* Rockefeller, YEOO concentration during laboratory bioassays varied between 1 and 75 mg/L. For the other strains, both the lowest and the highest concentrations varied and were adjusted accordingly. The lowest concentration varied between 4.2 mg/L (i.e. Caseara, Oiapoque, and Macapá) and 5 mg/L (i.e. Belo Horizonte), while the highest concentrations varied between 100 mg/L (i.e. Caseara and Macapá) and 150 mg/L (i.e. Oiapoque and Belo Horizonte). Mortality was registered after 24 h of larvicidal exposure at each concentration. This procedure was conducted at least three times for each strain in laboratory conditions at Laboratório de Bioquímica e Fisiologia de Insetos, at Fiocruz (LABFISI). All assays were carried out with two to three positive controls with the Rockefeller strain, presented as supplementary data (Additional file 1: Table S1). The lethal concentration data for the Rockefeller strain available in Workman et al*.* [27] were used as a reference for resistance ratio calculations.

### Bioassays under environmental conditions with natural light and temperature fluctuation

The toxicity assays of YEOO were also carried under environmental conditions with natural light and temperature fluctuation in the Department of Health station of Belo Horizonte (*Secretaria de Saúde da cidade de Belo Horizonte*) using *Ae. aegypti “*Belo Horizonte” (MG) and Rockefeller strains, the second as an internal control. These assays were performed between January and March 2019 (summertime) in an interior area of the building with partial sun exposure. Egg hatching and larval rearing under environmental conditions were carried out using identical insectary material (e.g. plastic trays, nets over breeding trays) and the same fish food from the standard insectary routine in the laboratory. We also used the adapted WHO protocol (i.e. 20–25 mosquito larvae, 24 h exposure, ten concentrations, multiple subsets per concentration, and at least three biological replicates). The lowest concentration was set to 5 mg/L and the highest concentration varied between 100 mg/L for the Rockefeller strain to 150 mg/L for the Belo Horizonte strain.

### Data analysis

Data from bioassays were organized by concentration in each biological replicate (results of technical subsets were combined for each assay). These data were used to create general linear models (GLM) with “logit” and “probit” models for each strain using the “glm” function in R version 4.0.1. For the Rockefeller and Belo Horizonte strains, the analysis was performed for two different conditions (i.e. laboratory and environmental). The intercept, slope, and quality indicators including residual, null deviance, residual deviance, and Akaike information criterion (AIC) of the model were provided by the summary created by the “glm” function. The pseudo R-squared for the GLM was calculated based on McFadden's pseudo-*R*^2^ [41] using the null deviance and residual deviance. Lethal concentrations and their confidence intervals at 95% were calculated using the R package “MASS.” YEOO larvicidal activity was evaluated using the larvicide screening criteria for EOs defined by Dias and Morais [24] (highly effective: LC_50_ < 50 mg/L; effective: LC_50_ < 100 mg/L; inactive: LC_50_ > 100 mg/L). The resistance ratio (RR) was computed using the Rockefeller strain in the corresponding condition as the reference, while ratios between conditions (RC) used laboratory assays as reference. Confidence intervals for ratios were calculated using the MOVER-R method [42] presented in the R package “pairwiseCI”. Plots were created using the R package “ggplot2.”

Further details on the quality and selection of the GLMs used to determine the lethal concentration are included in the supplementary data (Additional file 2: Text S1).

## Results

### Larvicide action under laboratory conditions

**Fig. 1:**
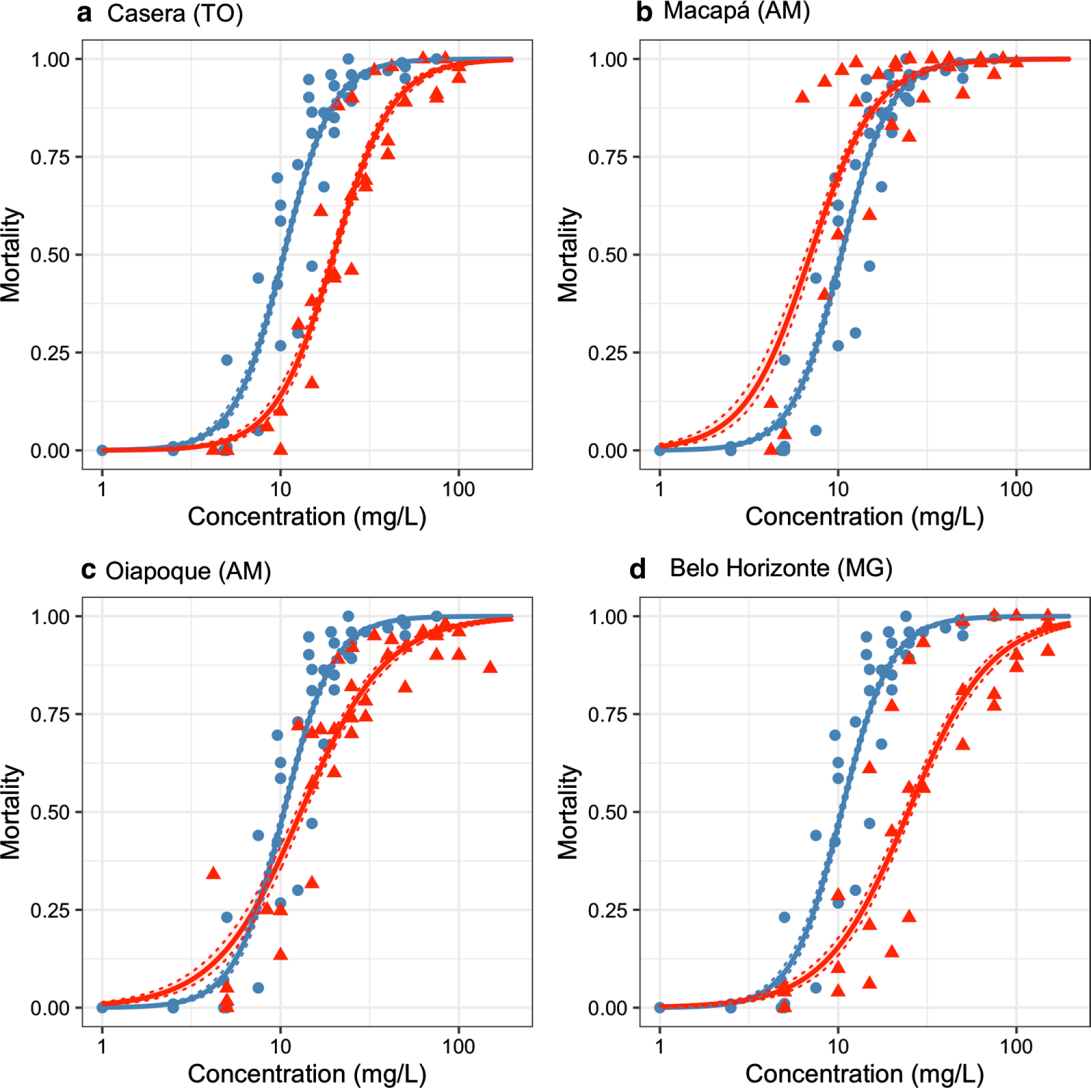
Larvicidal activity of YEOO against *Ae. aegypti* in laboratory conditions. The Rockefeller strain was used as reference (blue lines and dots). Red lines and triangles represent specific strains: **a** Caseara (TO); **b** Macapá (AM); **c** Oiapoque (AM); and **d** Belo Horizonte (MG). Dots/triangles: mortality of each concentration per assay. Continuous lines: graphic representation of GLMs. Dotted lines: a graphic description of confidence intervals of GLMs at 95%

YEOO was highly effective (LC_50_ < 50 mg/L) against all *Ae. aegypti* strains tested under laboratory conditions (Fig. 1 and Table 2). The highest LC_50_ in the laboratory was observed for Belo Horizonte (LC_50_ = 24.7 mg/L), presenting a value approximately half of the threshold for labeling botanical larvicidal activity as highly effective. The lethal concentrations for 90% and 95% mortality varied in the range of 17.7–79.7 mg/L and 24.3–118.9 mg/L.

**Table 2:** Lethal concentrations for YEOO against *Ae. aegypti* in laboratory conditions

Strain	LC_50_ (mg/L)	LC_90_ (mg/L)	LC_95_ (mg/L)
Rockefeller (reference)	10.4 (10.1–10.7)	21.0 (20.0–21.9)	26.6 (25.1–28.1)
Caseara	19.9 (19.1–20.6)	45.7 (42.8–48.7)	60.6 (55.9–65.8)
Macapá	7.0 (6.6–7.4)	17.7 (16.5–19.0)	24.3 (22.2–26.7)
Oiapoque	13.0 (12.3–13.8)	43.5 (39.9–47.4)	65.6 (58.6–73.5)
Belo Horizonte	24.7 (23.5–26.0)	79.7 (72.2–87.9)	118.9 (104.7–134.3)

LC_50–95_: lethal concentration at 50–95% mortality

The lethal concentrations for the Caseara and Belo Horizonte strains were significantly higher than the values for the Rockefeller strain according to the GLMs. In comparison, the lethal concentration for the Oiapoque strain was only higher than that for the Rockefeller strain above the LC_40_. For the Macapá strain, YEOO demonstrated higher larvicidal activity than for the Rockefeller strain up to LC_90_ (Fig. 1 and Additional file 3: Figure S1).

The GLM for the Rockefeller strain presented a higher slope (3.14) than GLMs from the four *Ae. aegypti* strains analyzed in this study (1.82–2.47; Additional file 2: Text S1). The lower slope in the Brazilian mosquito strains indicated higher heterogeneity in YEOO susceptibility between individuals, as expected for recently colonized strains. This heterogeneity may explain why resistance ratios tended to increase when using lethal concentrations from higher mortality values (Additional file 3: Figure S1).

The quality estimates for the GLMs indicated that the GLM for the Macapá strain data was less representative of the data than the GLMs for the other tested strains, and the interpretation of this result requires caution (Additional file 2: Text S1).

### Larvicidal action under environmental conditions

**Fig. 2:**
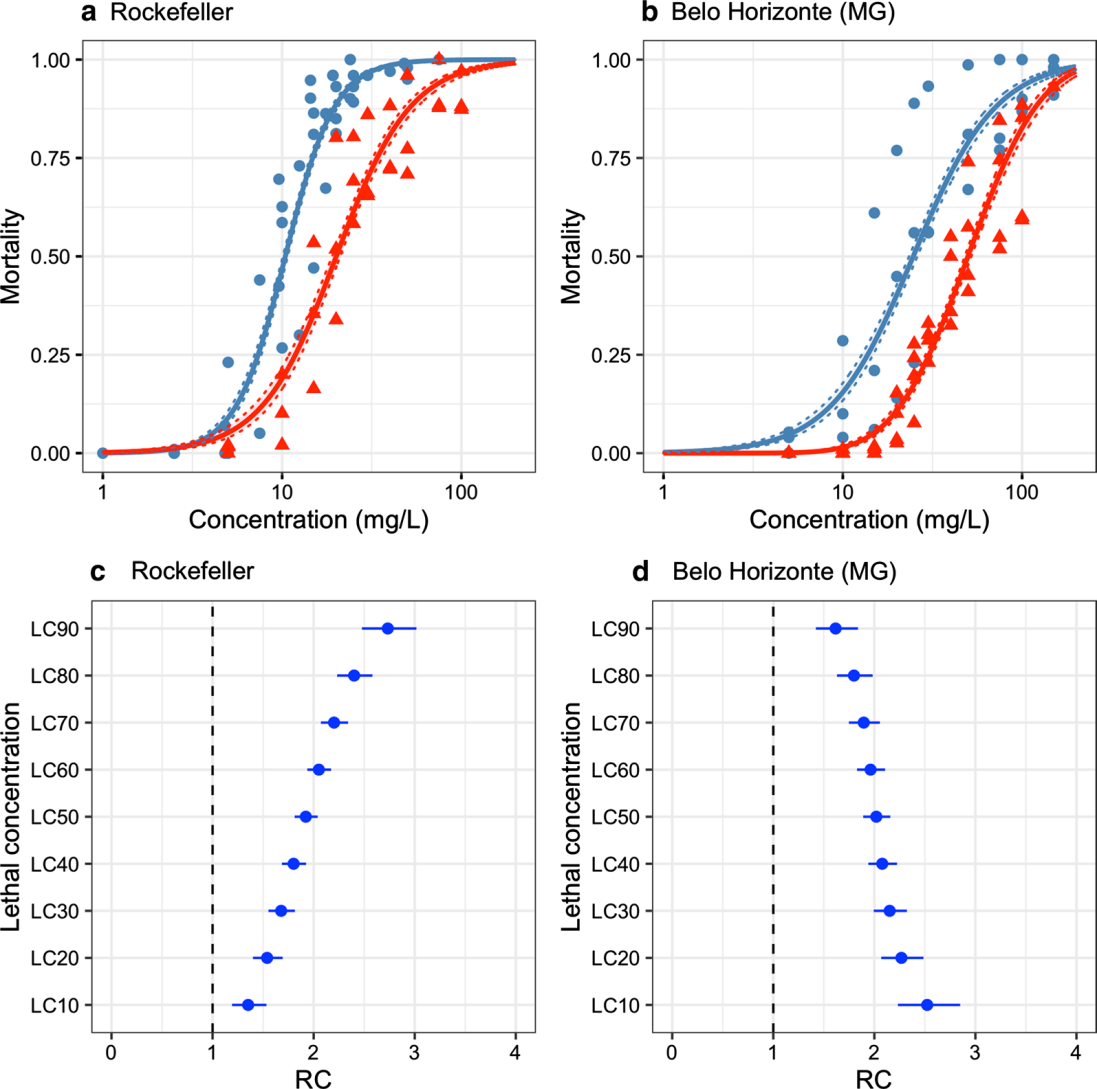
Larvicidal activity of YEOO against *Ae. aegypti* in different conditions. **a**, **b** GLMs for Rockefeller and Belo Horizonte, respectively; blue lines and dots: GLMs and data from assays in the laboratory. Red lines and triangles: GLMs and data from assays under environmental conditions. Dots/triangles: mortality for each concentration per assay. Continuous lines: a graphic representation of GLMs. Dotted lines: a graphic description of confidence intervals of GLMs at 95%. **c**, **d** Ratio between the lethal concentration of assays under environmental conditions and under laboratory conditions (RC = LC_E/LC_L). Blue dots: the ratio between environmental conditions and laboratory. Blue lines: confidence intervals at 95% calculated by MOVER-R method[42]

Assays to determine larvicidal activity were also carried under environmental conditions with natural light and temperature fluctuation for Rockefeller and Belo Horizonte strains. For both strains, lethal concentrations for assays under environmental conditions were significantly higher than the values of assays performed under laboratory conditions (Fig. 2). The ratio between conditions (RC, environmental vs. laboratory) varied between 1.35 and 2.73 for the Rockefeller strain, and between 1.63 and 2.48 for the Belo Horizonte strain.

The YEOO still displayed high effectiveness (LC_50_ < 50 mg/L) under environmental conditions, with an LC_50_ value slightly below the threshold for the Belo Horizonte strain (LC_50_ = 49.9 mg/L), and 20 mg/L for the Rockefeller strain (Table 3).

**Table 3:** Lethal concentration for YEOO against *Ae. aegypti* for assays in environmental conditions

Strain	Conditions	LC_50_ (mg/L)	LC_90_ (mg/L)	LC_95_ (mg/L)
Rockefeller	Laboratory	10.4 (10.1–10.7)	21.0 (20.0–21.9)	26.6 (25.1–28.1)
	Environmental	20.0 (19.0–21.1)	57.3 (52.5–62.6)	81.9 (73.2–91.7)
Belo Horizonte	Laboratory	24.7 (23.5–26.0)	79.7 (72.2–87.9)	118.6 (104.7–134.3)
	Environmental	49.9 (47.9–52.0)	128.8 (118.5–140.0)	168.5 (152.6–186.0)

*LC*_*50–95*_ lethal concentration at 50–95% mortality

### Resistance ratios

**Fig. 3:**
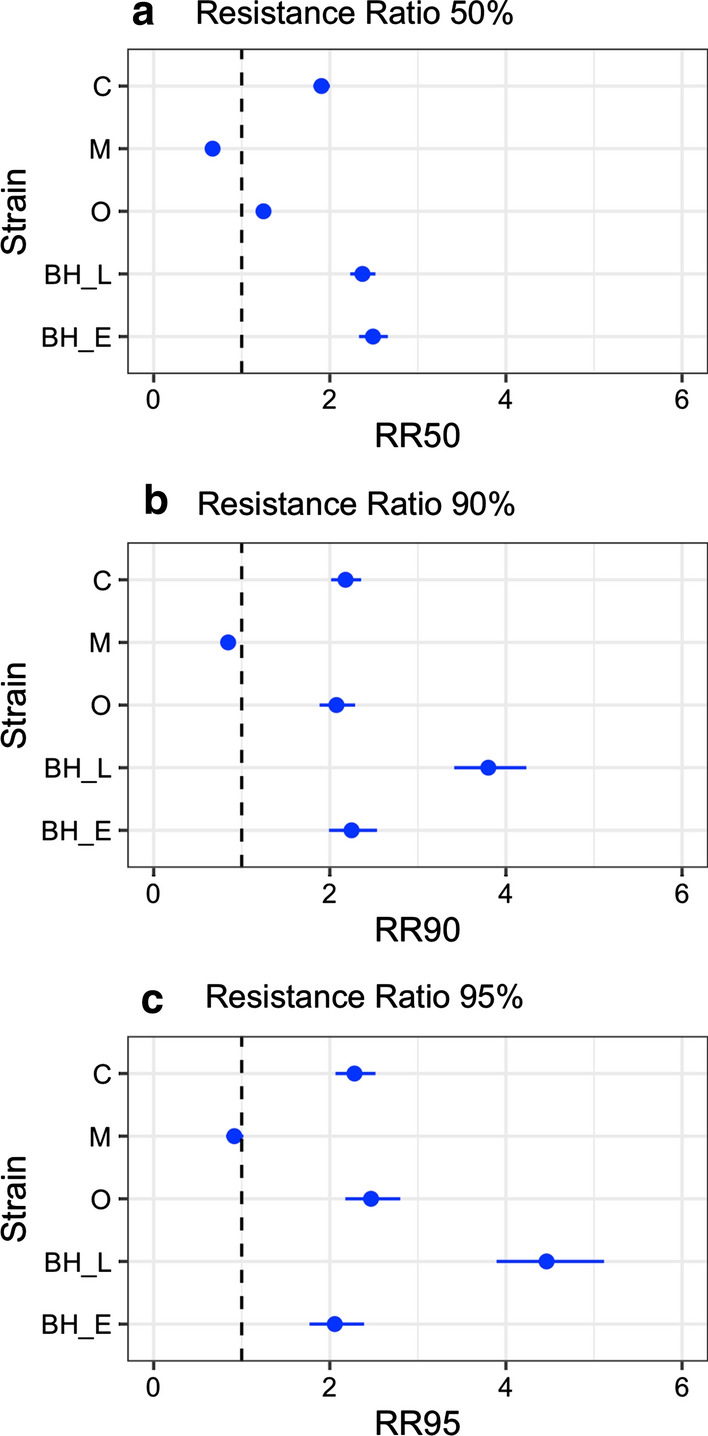
The resistance ratios in four *Ae. aegypti* strains for YEOO larvicidal activity. **a** Resistance ratios at 50% mortality; **b** resistance ratios at 90% mortality; **c** resistance ratios at 95% mortality. Blue dots: resistance ratios. Blue lines: confidence intervals at 95% calculated by the MOVER-R method [42]. C: assays from Caseara strain; M: assays from Macapá strain; O: assays from Oiapoque strain; BH_L: assays from Belo Horizonte strain carried out in laboratory conditions; BH_E: assays from Belo Horizonte carried out in environmental conditions with natural light and temperature fluctuation

According to the criteria proposed by WHO for neurotoxic chemical insecticides, for all RR values below 5, a mosquito strain can be labeled “susceptible” (Fig. 3). The RR for the Macapá strain (RR_50_ = 0.67, RR_90_ = 0.85, RR_95_ = 0.92) was below 1, with a lower/similar tolerance for the larvicide relative to the laboratory susceptibility reference. The RRs for the Belo Horizonte strain under environmental conditions (RR_50_ = 2.48, RR_90_ = 2.27, RR_95_ = 2.06) and the Caseara strain (RR_50_ = 1.91, RR_90_ = 2.18, RR_95_ = 2.28) showed similar values close to 2 for all mortality percentages. For the Oiapoque strain, the RR_50_ (1.25) was slightly above 1, but the resistance ratios for 90% and 95% mortality (RR_90_ = 2.08, RR_95_ = 2.47) increased to similar levels as the Belo Horizonte strain under environmental conditions and the Caseara strain. The highest resistance ratio values were observed in the Belo Horizonte strain under laboratory conditions for 90% and 95% mortality (RR_90_ = 3.80, RR_95_ = 4.46), while its resistance ratio at 50% was similar to assays carried under environmental conditions (RR_50_ = 2.37).

## Discussion

YEOO was a highly effective larvicide (LC_50_ < 50 mg/L, criteria defined by Dias and Morais [24]) for all Brazilian *Ae. aegypti* strains tested. These strains were collected in four different cities across three Brazilian states, indicating YEOO efficacy for mosquitoes of different geographic origins. This activity was consistent with our previous assessment using susceptible reference lineages (i.e. Rockefeller, Liverpool) in multiple laboratories [27]. The high larvicidal efficacy and low resistance ratios in mosquito strains with deltamethrin (PY) and temephos (OP) resistance suggest YEOO as a viable alternative tool against immature stages of mosquito populations with insecticide resistance.

The development of botanical insecticides for mosquito control requires risk evaluation assessments to identify the possibility of cross-resistance [19]. Here, we used Oiapoque and Macapá strains with known phenotypes for insecticide resistance (i.e. deltamethrin and temephos) and a well-characterized molecular mechanism for pyrethroid resistance (i.e. *kdr* alleles) [36, 37]. We recently collected a third resistant strain from a region well known for its history of insecticide resistance in *Ae. aegypti*, Belo Horizonte [35, 38]. The resistance ratios for 50%, 90%, and 95% mortality (RR_50–95_) for all strains were below 5. For most strains, the higher resistance ratios were approximately 2 for most mortality levels. The greatest exception was the Macapá strain, with the lowest RR_50–95_ (RR < 1). The resistance ratios for the Belo Horizonte strain were contradictory for the higher mortality values, since assays under laboratory conditions yielded higher RRs of close to 4 for RR_90_ and RR_95_. In contrast, activity under environmental conditions showed RRs of around 2 for all mortality ranges, similar to those seen with the Caseara strain. These differences in the Belo Horizonte resistance ratios can be explained by the age of the egg paper used in the laboratory bioassay. For these assays, we used an F3 generation of 5 to 6 months (ideally should be until 3 or 4 months), which may have impacted hatchability across egg papers [43]. This impact may have added variation among biological replicates that promoted increased heterogeneity and lowered the slope of assays with Belo Horizonte in the laboratory, which may explain the increased resistance ratios for higher lethal concentrations (RR_90–95_) when compared with RR_50_. For the other assays, we were able to use egg papers of less than 4 months. The similar results for the three resistant strains (RR_5095_ ≈ 2) and the susceptible field strain from Caseara, i.e. the lack of correlation between resistance ratios for temephos and deltamethrin and the resistance ratios for YEOO, indicated a low risk for cross-resistance between our product based on orange oil and these synthetic neurotoxic insecticides. Moreover, all resistance ratios indicated susceptibility to YEOO based on the criteria adopted for neurotoxic chemical insecticides (RR < 5: not resistant) [39]. However, the application of these criteria on EOs requires caution due to the different nature of the compounds (a pure neurotoxic compound with a clear mode of action versus multiple components with different effects in insects), and the potential for EOs exhibiting different behavior in the field from that of traditional insecticides.

For most botanical components, the mode of action as insecticide remains largely uncharacterized, with some studies suggesting a broad diversity of action modes for these secondary metabolites. In this study, the high mortality in 24 h assays and the fast-repetitive movement of larvae after contact with YEOO (Additional file 4: Video S1) also suggested a potential neurotoxic effect. Limonene, the main component of orange oil, has moderate/high anticholinesterase activity by inhibiting the enzyme acetylcholinesterase in insect synapses [44–47]. Other components of orange oil (i.e. gamma-terpinene) demonstrate similar neurotoxic effects. Moreover, alternative mechanisms may also play a role in YEOO, since YEOO ingestion is associated with severe midgut epithelial damage including destruction of microvilli, vacuolization of midgut cells, and damage to cell junctions and basal lamina [48]. Overall, botanical substances can promote multiple insecticidal effects. Synthetic insecticides share some of these effects with botanicals, such as the neurotoxic effects by inhibiting acetylcholinesterase, blocking receptors for GABA or octopamine, and stopping development due to insect growth regulator analogs. Still, some insecticidal modes of action in botanicals, as observed by Kelly et al. [48], are novel or rarely explored in control strategies, such as inhibition of mitochondrial respiration or detoxification enzymes, phototoxicity, generation of ROS, and midgut damage [26, 49, 50]. The use of these substances or the combination with other methods presents a potential alternative to reduce the impact of insecticide resistance [26]. For orange oil, it is unlikely that the mode of action targets the voltage-gated sodium channel (Na_V_) of the mosquito. Multiple *Kdr* alleles in Na_V_ (i.e. V1016I, F1534C) were found to cause pyrethroid resistance in *Ae. aegypti* in Brazil [35, 38], which may also impact other active substances targeting this molecule. Under this scenario, we would expect a loss of activity of orange oil in strains with high pyrethroid resistance (Table 1; Macapá, Oiapoque, Belo Horizonte), leading to higher resistance ratios than observed if YEOO targeted Na_V_. On the other hand, the limonene effect on anticholinesterase is not incompatible with high larvicidal activity in populations with temephos resistance. For temephos (OP), the overexpression of multiple detoxifying enzymes in *Ae. aegypti* is the resistance mechanism, which makes the information about resistance unrelated to the target molecule of organophosphates [15–18]. The studies about botanicals' modes of action as insecticides are costly and time-consuming, and only a few botanicals are already fully characterized. The pyrethrum extract (i.e. extract from *Chrysanthemum cinerariaefolium* flowers) is one of the primary examples of a well-characterized botanical insecticide, which allowed the development of synthetic pyrethroids (e.g. deltamethrin) based on pyrethrins, the main active components of this extract [21].

The YEOO was highly active under both tested scenarios. However, we identified a variation in larvicidal activity between laboratory conditions and environmental conditions with natural light and temperature fluctuation, as larvae that were bred and maintained under environmental conditions were approximately twice as tolerant to YEOO as laboratory-reared larvae, requiring a higher concentration to achieve similar mortality. It is possible that either the mosquito larvae reared under environmental conditions were healthier than those reared in the laboratory, or that YEOO degradation due to exposure to natural light and ambient temperature reduced larvicidal activity. The fast action of YEOO (high mortality at 24 h) and the 2-week stability suggested by preliminary semi-field trials [51] indicate that YEOO degradation is unlikely to explain the variations observed between the assays performed under laboratory and environmental conditions. For this reason, larvae maintained under natural light and summer temperature may have a physiological advantage, which would justify a higher tolerance for the larvicide. Mosquito larvae subjected to low-energy ultraviolet exposure (UVA) have demonstrated higher expression of detoxifying enzymes, making them more tolerant to various insecticides [52]. Larvae growing in sunlight should present a similar effect when compared with laboratory breeding without UV radiation. Moreover, our experiment was conducted in Belo Horizonte (Brazil) during summertime, a season with higher densities of *Ae. aegypti* mosquitoes [53, 54]. Faster growth may have resulted in the earlier onset of the fourth larval stage, which may have also influenced the larvicidal activity of YEOO [27]. We observed this tendency in two different strains with different colonization histories (i.e. Rockefeller, the reference strain maintained for decades in the laboratory, and the Belo Horizonte strain, with a maximum of three generations in the laboratory), which reinforces the importance of evaluating larvicidal activity under field conditions of light and temperature. The controlled environment of a laboratory was essential for the development and reproducibility of our studies. Still, translational research aiming to develop new control tools must include testing stages in non-controlled environments that are more faithful to vectors' natural habitats.

## Conclusion

Botanical insecticides are already a reality for agriculture pests. However, botanicals for mosquito control are still limited to a few repellent tools typically based on citronella and lemon eucalyptus [26]. The development of botanical insecticides is often a long process that requires regulatory approvals [19]. Here, we demonstrated that YEOO is a feasible approach for controlling *Ae. aegypti* in environmental conditions with natural light and temperature fluctuations. The larvicidal activity of YEOO against strains resistant to deltamethrin (PY) and temephos (OP) suggests its potential as an alternative to traditional synthetic insecticides in IVM programs. However, this larvicide still requires additional studies to meet the requirements for commercialization, particularly with regard to the impact on non-target aquatic organisms and effectiveness against *Ae. aegypti* in natural breeding sites (i.e. field testing).

## Supplementary Information

**Additional file 1: Table S1.** Mortality of positive control in laboratory assays with YEOO against reference strain *Aedes aegypti*—Rockefeller.

**Additional file 2: Text S1.** Quality and selection of General linear models to determine lethal concentration.

**Additional file 3: Figure S1.** The resistance ratios for four strains in laboratory conditions for YEOO larvicidal activity. RR: resistance ratio; Blue dots: resistance ratios; Blue lines: confidence intervals at 95% calculated by MOVER-R method [42].

**Additional file 4: Video S1.** Larvae behavior after 30 min contact with YEOO larvicide. This video was recorded by a mobile phone LG K1+ and edited by the software iMovie. The 22 s movie shows the behavior of five larvae of *Ae.* aegypti at stage L3, after the contact with YEOO at 30 mg/L for 30 min.

## Data Availability

All data generated or analysed during this study are included in this published article [and its Additional files].

## References

[1] Pan American Health Organization (PAHO). Handbook for Integrated Vector Management in the Americas. Washington, D.C.: PAHO; 2019.

[2] Achee NL, Grieco JP, Vatandoost H, Seixas G, Pinto J, Ching-NG L (2019). Alternative strategies for mosquito-borne arbovirus control. PLoS Negl Trop Dis.

[3] Nájera JA, González-Silva M, Alonso PL (2011). Some lessons for the future from the Global Malaria Eradication Programme (1955–1969). PLoS Med.

[4] Hemingway J, Ranson H (2000). Insecticide resistance in insect vectors of Human disease. Annu Rev Entomol.

[5] Yu SJ (2015). The toxicology and biochemistry of insecticides.

[6] Dusfour I, Vontas J, David J-P, Weetman D, Fonseca DM, Corbel V (2019). Management of insecticide resistance in the major *Aedes* vectors of arboviruses: advances and challenges. PLoS Negl Trop Dis.

[7] Brady OJ, Hay SI (2020). The global expansion of dengue: How *Aedes aegypti* mosquitoes enabled the first pandemic arbovirus. Annu Rev Entomol.

[8] Burt FJ, Chen W, Miner JJ, Lenschow DJ, Merits A, Schnettler E (2017). Chikungunya virus: an update on the biology and pathogenesis of this emerging pathogen. Lancet Infect Dis.

[9] Kindhauser MK, Allen T, Frank V, Santhana RS, Dye C (2016). Zika: the origin and spread of a mosquito-borne virus. Bull World Health Organ.

[10] World Health Organization (WHO). Global health estimates 2020: disease burden by cause, age, sex, by country and by region, 2000–2019. Geneva; 2020.

[11] Moyes CL, Vontas J, Martins AJ, Ng LC, Koou SY, Dusfour I (2017). Contemporary status of insecticide resistance in the major *Aedes* vectors of arboviruses infecting humans. PLoS Negl Trop Dis.

[12] Chen M, Du Y, Wu S, Nomura Y, Zhu G, Zhorov BS (2019). Molecular evidence of sequential evolution of DDT- and pyrethroid-resistant sodium channel in *Aedes aegypti*. PLoS Negl Trop Dis.

[13] Du Y, Nomura Y, Zhorov B, Dong K (2016). Sodium channel mutations and pyrethroid resistance in *Aedes aegypti*. Insects.

[14] Cosme LV, Gloria-Soria A, Caccone A, Powell JR, Martins AJ (2020). Evolution of *kdr* haplotypes in worldwide populations of *Aedes aegypti*: Independent origins of the F1534C *kdr* mutation. PLoS Negl Trop Dis.

[15] Strode C, de Melo-Santos M, Magalhães T, Araújo A, Ayres C (2012). Expression profile of genes during resistance reversal in a temephos selected strain of the dengue vector, *Aedes aegypti*. PLoS ONE.

[16] Diniz DFA, de Melo-Santos MAV, de Santos EM, Beserra EB, Helvecio E, de Carvalho-Leandro D (2015). Fitness cost in field and laboratory *Aedes aegypti* populations associated with resistance to the insecticide temephos. Parasit Vectors..

[17] Poupardin R, Srisukontarat W, Yunta C, Ranson H, Bhatt S, Gething P (2014). Identification of carboxylesterase genes implicated in temephos resistance in the dengue vector *Aedes aegypti*. PLoS Negl Trop Dis.

[18] Grisales N, Poupardin R, Gomez S, Fonseca-Gonzalez I, Ranson H, Lenhart A (2013). Temephos resistance in *Aedes aegypti* in Colombia compromises dengue vector control. PLoS Negl Trop Dis.

[19] World Health Organization (WHO), Food and Agriculture Organization of the United Nations (FAO). Guidelines for the registration of microbial, botanical and semiochemical pest control agents for plant protection and public health uses. Yadav/WHOPES DR, editor. Rome; 2017.

[20] Vieira PC, Fernandes JP, Andrei CC. Plantas inseticidas. In: Simões CMO, Schenkel EP, Gosmann G, de Mello JCP, Mentz LA, Petrovick PR, editors. Farmacognosia da planta ao Medicamento. 4th ed. Porto Alegre/Florianopolis: Ed. UFRGS/ Ed. UFSC; 2002. p. 751–66.

[21] Matsuo N (2019). Discovery and development of pyrethroid insecticides. Proc Japan Acad Ser B.

[22] Rehman JU, Ali A, Khan IA (2014). Plant based products: use and development as repellents against mosquitoes: a review. Fitoterapia.

[23] Simões CMO, Spitzer V. Óleos voláteis. In: Simões CMO, Schenkel EP, Gosmann G, Mello JCP de, Mentz LA, Petrovick PR, editors. Farmacognosia da planta ao Medicamento. 4th ed. Porto Alegre/Florianopolis: Ed. UFRGS/ Ed. UFSC; 2002. p. 397–425.

[24] Dias CN, Moraes DFC (2014). Essential oils and their compounds as *Aedes aegypti* L. (Diptera: Culicidae) larvicides: review. Parasitol Res.

[25] Pavela R (2015). Essential oils for the development of eco-friendly mosquito larvicides: a review. Ind Crops Prod.

[26] Pavela R, Maggi F, Iannarelli R, Benelli G (2019). Plant extracts for developing mosquito larvicides: from laboratory to the field, with insights on the modes of action. Acta Trop.

[27] Workman MJ, Gomes B, Weng J-L, Ista LK, Jesus CP, David MR (2020). Yeast-encapsulated essential oils: a new perspective as an environmentally friendly larvicide. Parasit Vectors.

[28] Bishop JR, Nelson G, Lamb J (1998). Microencapsulation in yeast cells. J Microencapsul.

[29] Souza RS, Diaz-Albiter HM, Dillon VM, Dillon RJ, Genta FA (2016). Digestion of yeasts and beta-1,3-glucanases in mosquito larvae: physiological and biochemical considerations. PLoS ONE.

[30] Souza RS, do Gama VFM, Schama R, Lima JBP, Diaz-Albiter HM, Genta FA (2019). Biochemical and functional characterization of glycoside hydrolase family 16 genes in *Aedes aegypti*larvae: identification of the major digestive β-1,3-glucanase. Front Physiol..

[31] Souza RS, Virginio F, Riback TIS, Suesdek L, Barufi JB, Genta FA (2019). Microorganism-based larval diets affect mosquito development, size and nutritional reserves in the yellow fever mosquito *Aedes aegypti* (Diptera: Culicidae). Front Physiol.

[32] Murugan K, Mahesh Kumar P, Kovendan K, Amerasan D, Subrmaniam J, Hwang J-S (2012). Larvicidal, pupicidal, repellent and adulticidal activity of *Citrus sinensis* orange peel extract against *Anopheles stephensi*, *Aedes aegypti* and *Culex quinquefasciatus* (Diptera: Culicidae). Parasitol Res.

[33] Warikoo R, Ray A, Sandhu JK, Samal R, Wahab N, Kumar S (2012). Larvicidal and irritant activities of hexane leaf extracts of *Citrus sinensis* against dengue vector *Aedes aegypti* L. Asian Pac J Trop Biomed.

[34] Cavalcanti ESB, de Morais SM, Lima MAA, Santana EWP (2004). Larvicidal Activity of essential oils from Brazilian plants against *Aedes aegypti* L. Mem Inst Oswaldo Cruz.

[35] Valle D, Bellinato DF, Viana-Medeiros PF, Lima JBP, de Martins-Junior AJ (2019). Resistance to temephos and deltamethrin in *Aedes aegypti* from Brazil between 1985 and 2017. Mem Inst Oswaldo Cruz..

[36] Salgueiro P, Restrepo-Zabaleta J, Costa M, Galardo AKR, Pinto J, Gaborit P (2019). Liaisons dangereuses: cross-border gene flow and dispersal of insecticide resistance-associated genes in the mosquito *Aedes aegypti* from Brazil and French Guiana. Mem Inst Oswaldo Cruz.

[37] de Sá ELR, de Rodovalho CM, de Sousa NPR, de Sá ILR, Bellinato DF, dos Dias LS (2019). Evaluation of insecticide resistance in *Aedes aegypti* populations connected by roads and rivers: the case of Tocantins state in Brazil. Mem Inst Oswaldo Cruz.

[38] Melo Costa M, Campos KB, Brito LP, Roux E, Melo Rodovalho C, Bellinato DF (2020). *Kdr* genotyping in *Aedes aegypti* from Brazil on a nation-wide scale from 2017 to 2018. Sci Rep.

[39] World Health Organization (WHO). Monitoring and managing insecticide resistance in *Aedes* mosquito populations: interim guidance for entomologists. WHO; 2016.

[40] World Health Organization (WHO). Guidelines for laboratory and field testing of mosquito larvicides. WHO; 2005.

[41] McFadden D, Zarembka P (1974). Conditional logit analysis of qualitative choice behavior. Frontiers in econometrics.

[42] Newcombe RG (2016). MOVER-R confidence intervals for ratios and products of two independently estimated quantities. Stat Methods Med Res.

[43] Brown HE, Smith C, Lashway S (2016). Influence of the length of storage on *Aedes aegypti* (Diptera: Culicidae) egg viability. J Med Entomol.

[44] Seo S-M, Kim J, Kang J, Koh S-H, Ahn Y-J, Kang K-S (2014). Fumigant toxicity and acetylcholinesterase inhibitory activity of 4 Asteraceae plant essential oils and their constituents against Japanese termite (*Reticulitermes speratus* Kolbe). Pestic Biochem Physiol.

[45] Seo S-M, Jung C-S, Kang J, Lee H-R, Kim S-W, Hyun J (2015). Larvicidal and acetylcholinesterase inhibitory activities of apiaceae plant essential oils and their constituents against *Aedes albopictus* and formulation development. J Agric Food Chem.

[46] Hostettmann K, Borloz A, Urbain A, Marston A (2006). Natural product inhibitors of acetylcholinesterase. Curr Org Chem.

[47] Mitsuo M, Watanabe H, Hiromu K (1997). Inhibition of acetylcholinesterase activity by monoterpenoids with a p-Menthane skeleton. J Agric Food Chem.

[48] Kelly P. (manuscript submitted).

[49] Jankowska M, Rogalska J, Wyszkowska J, Stankiewicz M, Jankowska M, Rogalska J (2017). Molecular targets for components of essential oils in the insect nervous system—a review. Molecules.

[50] Lee S-H, Ha KB, Park DH, Fang Y, Kim JH, Park MG (2018). Plant-derived compounds regulate formation of the insect juvenile hormone receptor complex. Pestic Biochem Physiol.

[51] Brant F das GC. Teste do óleo essencial de laranja (*Citrus sinensis*) encapsulado em leveduras para o controle da população de *Aedes aegypti* em Belo Horizonte—MG. Fundação Oswaldo Cruz; 2019.

[52] Tetreau G, Chandor-Proust A, Faucon F, Stalinski R, Akhouayri I, Prud’homme SM (2013). Contrasting patterns of tolerance between chemical and biological insecticides in mosquitoes exposed to UV-A. Aquat Toxicol.

[53] Camara DCP, Codeço CT, Juliano SA, Lounibos LP, Riback TIS, Pereira GR (2016). Seasonal differences in density but similar competitive impact of Aedes albopictus (Skuse) on *Aedes aegypti* (L.) in Rio de Janeiro, Brazil. PLoS ONE.

[54] de Custódio JMO, Nogueira LMS, Souza DA, Fernandes MF, Oshiro ET, de Oliveira EF (2019). Abiotic factors and population dynamic of *Aedes aegypti* and *Aedes albopictus* in an endemic area of dengue in Brazil. Rev Inst Med Trop Sao Paulo..

