# Supplementary material for: High larvicidal efficacy of yeast-encapsulated orange oil against Aedes aegypti strains from Brazil
Source: Parasit Vectors. 2021 May 22;14:272. doi: 10.1186/s13071-021-04733-2 (PMC8140510; doi:10.1186/s13071-021-04733-2)
Supplement: Supplementary file 1 — Additional file 1: Table S1. Mortality of positive control in laboratory assays with YEOO against reference strain Aedes aegypti—Rockefeller. [file 13071_2021_4733_MOESM1_ESM.docx]

Table S1. Mortality of positive control in laboratory assays with YEOO against reference strain *Aedes aegypti* - Rockefeller

| Assays | Biological  Replicates | Concentration  (mg/L) | Mortality | | |
| --- | --- | --- | --- | --- | --- |
|  |  |  | Mean | Min | Max |
| Belo Horizonte | 3 | 10 | 0.480 | 0.010 | 0.919 |
| Belo Horizonte | 3 | 20 | 0.829 | 0.646 | 0.990 |
| Belo Horizonte | 3 | 30 | 0.916 | 0.828 | 1.000 |
| Oiapoque | 4 | 8.4 – 10 | 0.333 | 0.010 | 0.529 |
| Oiapoque | 4 | 16.8 – 20 | 0.607 | 0.530 | 0.850 |
| Oiapoque | 4 | 24.4 – 30 | 0.722 | 0.600 | 0.920 |
| Caseara | 3 | 8.4 – 10 | 0.147 | 0.000 | 0.430 |
| Caseara | 3 | 16.8 – 20 | 0.697 | 0.550 | 0.950 |
| Caseara | 3 | 24.4 – 28 | 0.803 | 0.600 | 1.000 |
| Macapá | 3 | 10 | 0.528 | 0.430 | 0.626 |
| Macapá | 3 | 16 – 20 | 0.846 | 0.820 | 0.863 |
| Macapá | 3 | 25 – 28 | 0.940 | 0.900 | 0.990 |
