# Supplementary material for: High larvicidal efficacy of yeast-encapsulated orange oil against Aedes aegypti strains from Brazil
Source: Parasit Vectors. 2021 May 22;14:272. doi: 10.1186/s13071-021-04733-2 (PMC8140510; doi:10.1186/s13071-021-04733-2)
Supplement: Supplementary file 2 — Additional file 2: Text S1. Quality and selection of General linear models to determine lethal concentration. [file 13071_2021_4733_MOESM2_ESM.docx]

**Text S2. Quality and selection of general linear models to determine lethal concentration**

1. Basic information quality and selection of GLMs

- General linear models (“glm” function)

All models were created by “glm” function in R version 4.0.1. For more information, you should verify R documentation about this function:

<https://www.rdocumentation.org/packages/stats/versions/3.6.2/topics/glm>

- Deviance estimates:

The deviance indicates the extent to which the likelihood of the saturated model exceeds the likelihood of the proposed model. A small value for deviance indicates that the proposed model has a good fit, while higher deviance values suggest a bad fit of the proposed model.

**Residuals**: estimates that indicate differences between observed and predicted values of the model.

**Null deviance**: deviance estimate for the null-model (*i.e.* intercept-only model).

**Residual deviance**: deviance estimate from the proposed model (A low residual deviance implies that the model is appropriate).

- McFadden's pseudo-𝑅^2^

The model estimates from a logistic regression are maximum likelihood estimates calculated through an iterative process. For this reason, equivalent statistic of R-squared cannot infer goodness-of-fit from the models. However, several authors developed "pseudo" R-squared, ranging from 0 to 1 with higher values indicating better model fit, to infer the quality of logistic regression models. Here, we use McFadden's pseudo-𝑅^2^ to support the model selection. This parameter can be easily calculated based on the deviance estimates provided by R.

$$McFadden^{'}s pseudo-R^{2}=1-\frac{Residual deviance}{Null deviance}$$

To a better understanding of this topic, we recommend the consultation of UCLA FAQ statistical page:

<https://web.archive.org/web/20130701052120/http://www.ats.ucla.edu:80/stat/mult_pkg/faq/general/Psuedo_RSquareds.htm>

- Akaike’s Information Criteria (AIC)

The AIC is an information-theoretical measure that describes the quality of a model. A model with low AIC is characterized by low complexity and a good fit.

1. Quality and selection of GLM

We generated two GLMs for each condition based on “logit” and “probit” binomial models. The models based on logit displayed lower AIC and higher pseudo-R^2^ in most conditions (six out of seven conditions) indicating a better representation of our data than models with probit. The model probit performed better only for assays using the Belo Horizonte strain during semi-field conditions (Table S1). We decided to only use the best quality model for each condition. Moreover, the GLM for the Macapá strain displayed higher residual deviances (789-824) than models from other strains (residual deviance: 209-413; average = 305). These estimates indicate that for the Macapá strain the GLM is less representative of the data, and it should be used with caution when interpreting the results. There is a higher divergence among replicates in this strain when compared to the other data sets, which suggest that other factor not evaluated here may be influencing this result.

| Strain | Cond | GLM | Slope | Wald test | | | Inter | Wald test | | |  | Deviance residuals | | | | |  | Dispersion parameter | | Pseudo  R^2^ | AIC |
| --- | --- | --- | --- | --- | --- | --- | --- | --- | --- | --- | --- | --- | --- | --- | --- | --- | --- | --- | --- | --- | --- |
|  |  |  |  | Std. | z | Sig. |  | Std. | z | Sig. |  | Min | 1Q | Med | 3Q | Max |  | Null  deviance | Residual  deviance |  |  |
| Rockefeller | Lab | **logit** | **3.14** | **0.10** | 31.67 | **<2e^-16^** | **-7.37** | **0.25** | -29.24 | **<2e^-16^** |  | **-6.88** | **-1.76** | **-0.26** | **2.25** | **5.31** |  | **3072.77** | **394.73** | **0.87** | **535.24** |
|  |  | probit | 1.76 | 0.05 | 35.03 | <2e^-16^ | -4.12 | 0.13 | -31.95 | <2e^-16^ |  | -6.60 | -1.78 | -0.43 | 2.26 | 5.19 |  | 3072.77 | 410.58 | 0.87 | 551.09 |
|  | UC | **logit** | **2.09** | **0.09** | **24.08** | **<2e^-16^** | **-6.27** | **0.28** | **-22.45** | **<2e^-16^** |  | **-5.24** | **-2.18** | **-1.18** | **1.18** | **6.30** |  | **1289.09** | **231.72** | **0.82** | **349.84** |
|  |  | probit | 1.19 | 0.04 | 26.82 | <2e^-16^ | -3.60 | 0.15 | -24.73 | <2e^-16^ |  | -5.43 | -2.30 | -0.85 | 1.42 | 6.42 |  | 1289.09 | 241.29 | 0.81 | 359.41 |
| Caseara | Lab | **logit** | **2.64** | **0.10** | **27.22** | **<2e^-16^** | **-7.88** | **0.30** | **-25.89** | **<2e^-16^** |  | **-5.51** | **-2.28** | **-1.23** | **1.82** | **7.39** |  | **1945.63** | **287.94** | **0.85** | **394.58** |
|  |  | probit | 1.49 | 0.05 | 30.48 | <2e^-16^ | -4.45 | 0.16 | -28.68 | <2e^-16^ |  | -5.71 | -1.99 | -1.11 | 1.52 | 7.57 |  | 1945.63 | 297.44 | 0.85 | 404.08 |
| Macapá | Lab | **logit** | **2.36** | **0.10** | **23.24** | **<2e^-16^** | **-4.57** | **0.24** | **-19.01** | **<2e^-16^** |  | **-7.27** | **-3.17** | **0.75** | **2.60** | **9.71** |  | **1552.6** | **556.4** | **0.64** | **637.62** |
|  |  | probit | 1.23 | 0.05 | 24.25 | <2e^-16^ | -2.36 | 0.13 | -18.58 | <2e^-16^ |  | -8.00 | -3.74 | 0.47 | 3.22 | 9.36 |  | 1552.63 | 597.24 | 0.62 | 678.46 |
| Oiapoque | Lab | **logit** | **1.82** | **0.07** | **25.78** | **<2e^-16^** | **-4.66** | **0.21** | **-22.41** | **<2e^-16^** |  | **-5.69** | **-2.44** | **-0.57** | **1.16** | **5.97** |  | **1386.40** | **274.86** | **0.80** | **412.33** |
|  |  | probit | 1.01 | 0.04 | 27.91 | <2e^-16^ | -2.57 | 0.11 | -23.55 | <2e^-16^ |  | -6.10 | -2.28 | -0.26 | 1.52 | 5.41 |  | 1386.40 | 303.41 | 0.78 | 440.88 |
| Belo  Horizonte | Lab | **logit** | **1.88** | **0.07** | **26.30** | **<2e^-16^** | **-6.02** | **0.24** | **-25.27** | **<2e^-16^** |  | **-5.74** | **-2.65** | **-0.47** | **2.53** | **6.95** |  | **1697.98** | **409.48** | **0.76** | **521.31** |
|  |  | probit | 1.08 | 0.04 | 29.55 | <2e^-16^ | -3.47 | 0.12 | -27.99 | <2e^-16^ |  | -5.82 | -2.34 | -0.19 | 2.36 | 7.05 |  | 1697.98 | 412.89 | 0.76 | 524.72 |
|  | UC | logit | 2.28 | 0.08 | 28.28 | <2e^-16^ | -8.92 | 0.30 | -29.26 | <2e^-16^ |  | -5.08 | -2.17 | -0.82 | 1.14 | 4.82 |  | 1658.96 | 208.87 | 0.87 | 347.19 |
|  |  | **probit** | **1.35** | **0.04** | **30.77** | **<2e^-16^** | **-5.29** | **0.16** | **-32.07** | **<2e^-16^** |  | **-4.94** | **-1.80** | **-0.38** | **1.09** | **4.88** |  | **1658.96** | **190.34** | **0.89** | **328.66** |

**Table S2. Regression parameters of generalized linear models and quality estimates.**

Cond: the condition of the assay; Lab: assays carried in laboratory conditions; UC: assays carried in uncontrolled conditions with natural light; Std: standard deviation; Inter: Intercept; Min: residual with the lower value; 1Q: 1^st^ quartile equivalent to 25%; Med: Median; 3Q: 3^rd^ quartile equivalent to 75%; Max: residual with the highest value; Pseudo-R^2^: Pseudo R-square for GLM calculated based on McFadden's pseudo-𝑅^2^; AIC: Akaike’s Information Criteria.
