# Supplementary figures and images for: High larvicidal efficacy of yeast-encapsulated orange oil against Aedes aegypti strains from Brazil
Source: Parasit Vectors. 2021 May 22;14:272. doi: 10.1186/s13071-021-04733-2 (PMC8140510; doi:10.1186/s13071-021-04733-2)

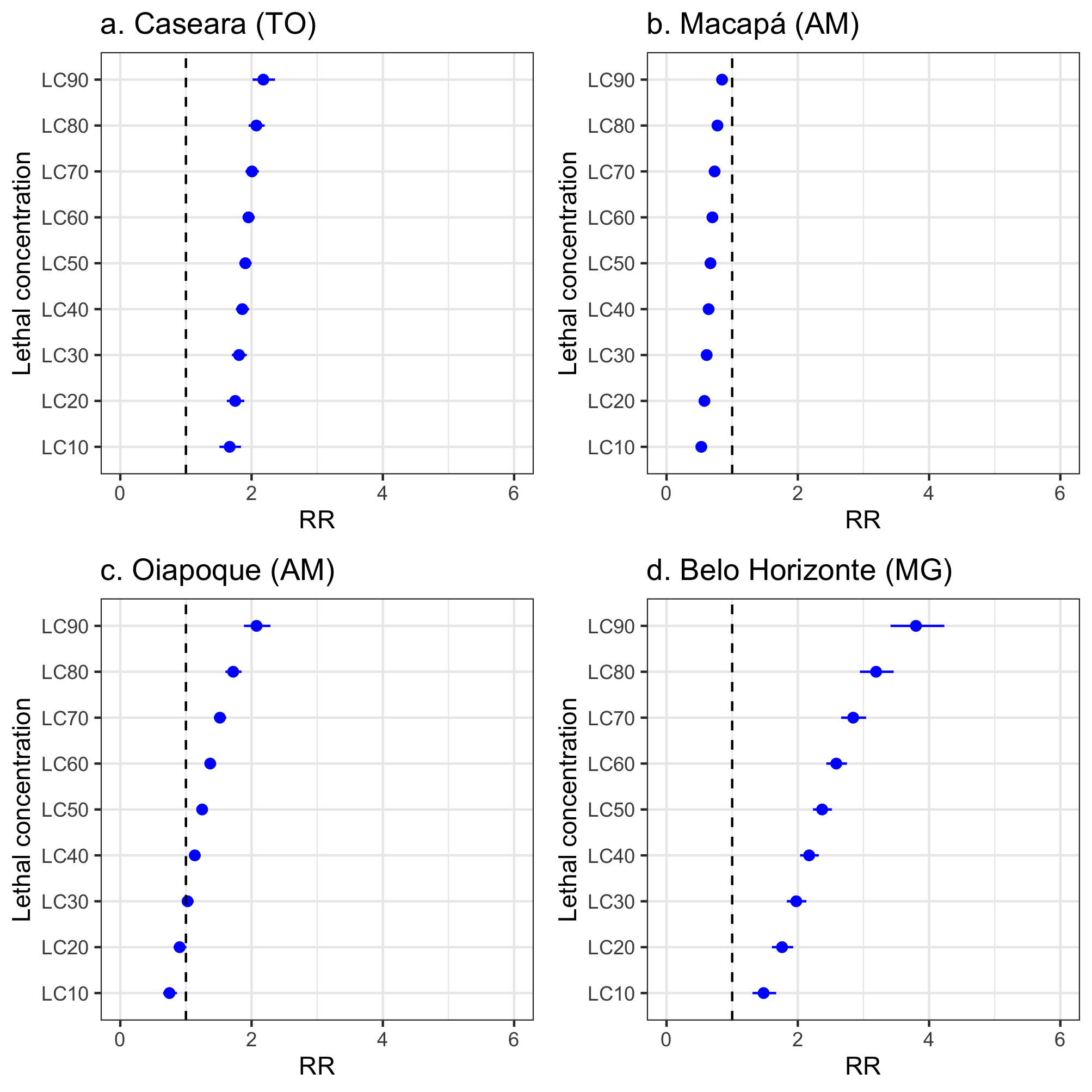

Supplement: Supplementary file 3 — Additional file 3: Figure S1. The resistance ratios for four strains in laboratory conditions for YEOO larvicidal activity. RR: resistance ratio; Blue dots: resistance ratios; Blue lines: confidence intervals at 95% calculated by MOVER-R method [42]. [file 13071_2021_4733_MOESM3_ESM.tiff]
